# Effects of a humanized CD47 antibody and recombinant SIRPα proteins on triple negative breast carcinoma stem cells

**DOI:** 10.3389/fcell.2024.1356421

**Published:** 2024-03-01

**Authors:** Sukhbir Kaur, Bianca Reginauld, Sam Razjooyan, Trung Phi, Satya P. Singh, Thomas J. Meyer, Margaret C. Cam, David D. Roberts

**Affiliations:** ^1^ Laboratory of Pathology, Center for Cancer Research, National Cancer Institute, National Institutes of Health, Bethesda, MD, United States; ^2^ Inflammation Biology Section, Laboratory of Molecular Immunology, National Institute of Allergy and Infectious Diseases, National Institutes of Health, Bethesda, MD, United States; ^3^ CCR Collaborative Bioinformatics, Resource, Office of Science and Technology Resources, National Cancer Institute, National Institutes of Health, Bethesda, MD, United States

**Keywords:** CD47, SIRPα, EMT, cancer stem cells, breast cancer

## Abstract

Signal regulatory protein-α (SIRPα, SHPS-1, CD172a) expressed on myeloid cells transmits inhibitory signals when it engages its counter-receptor CD47 on an adjacent cell. Elevated CD47 expression on some cancer cells thereby serves as an innate immune checkpoint that limits phagocytic clearance of tumor cells by macrophages and antigen presentation to T cells. Antibodies and recombinant SIRPα constructs that block the CD47-SIRPα interaction on macrophages exhibit anti-tumor activities in mouse models and are in ongoing clinical trials for treating several human cancers. Based on prior evidence that engaging SIRPα can also alter CD47 signaling in some nonmalignant cells, we compared direct effects of recombinant SIRPα-Fc and a humanized CD47 antibody that inhibits CD47-SIRPα interaction (CC-90002) on CD47 signaling in cancer stem cells derived from the MDA-MB- 231 triple-negative breast carcinoma cell line. Treatment with SIRPα-Fc significantly increased the formation of mammospheres by breast cancer stem cells as compared to CC-90002 treatment or controls. Furthermore, SIRPα-Fc treatment upregulated mRNA and protein expression of ALDH1 and altered the expression of genes involved in epithelial/mesenchymal transition pathways that are associated with a poor prognosis and enhanced metastatic activity. This indicates that SIRPα-Fc has CD47-mediated agonist activities in breast cancer stem cells affecting proliferation and metastasis pathways that differ from those of CC-90002. This SIRPα-induced CD47 signaling in breast carcinoma cells may limit the efficacy of SIRPα decoy therapeutics intended to stimulate innate antitumor immune responses.

## 1 Introduction

CD47 is a widely expressed integral membrane protein that serves as a counter receptor for signal regulatory protein-α (SIRPα), which is highly expressed on phagocytes and other myeloid lineages. Binding to CD47 triggers inhibitory signaling through SIRPα that prevents macrophage phagocytosis of nonmalignant and cancer cells (Oldenborg et al., 2000; Jaiswal et al., 2009; Willingham et al., 2012; Barclay and Van den Berg, 2014). Many studies have reported increased expression of CD47 on neoplastic cells and associations of higher tumor CD47 expression with decreased survival (Willingham et al., 2012; Barclay and Van den Berg, 2014; Gholiha et al., 2022). These studies generally focus on the role of CD47 as a passive counter-receptor for SIRPα, which protects tumor cells from phagocytic clearance or antigen presentation via SIRPα-expressing macrophages, neutrophils and dendritic cells (Gardai et al., 2005; Chao et al., 2010; Willingham et al., 2012; Matlung et al., 2017). Therapeutic antibodies and a SIRPα-Fc fusion decoy designed to block the interaction between CD47 and SIRPα have entered multiple clinical trials and provided anecdotal evidence for efficacy in some cancers (Advani et al., 2018; Sikic et al., 2019; Ansell et al., 2021; Querfeld et al., 2021; Son et al., 2022; Zeidan et al., 2022).

CD47 is also a signaling receptor for the secreted matricellular protein thrombospondin-1 (TSP1) (Kaur et al., 2021). CD47 has multiple signaling functions in nonmalignant cells (Roberts et al., 2015). TSP1 binding induces CD47 signaling that regulates growth factor receptors, cell fate, viability, and responses to cellular stresses such as radiation and chemotherapy (Kaur et al., 2021). Studies of the crosstalk between T cells and dendritic cells indicate that SIRPα binding can also induce CD47 signaling (Latour et al., 2001; Sarfati et al., 2008). Some of these CD47 signaling functions may be maintained or co-opted by malignant cancer cells. Thus, in addition to blocking interactions with phagocytes, binding of SIRPα-Fc fusion decoys could potentially alter CD47 signaling in tumor cells.

In addition to blocking the binding of SIRPα and/or TSP1 to CD47, some CD47 antibodies have agonist activities that alter CD47 signaling in the absence of its physiological ligands (Kaur et al., 2020). This could involve allosteric effects of antibody binding on CD47 signaling, antibody-induced dimerization of CD47, or perturbation of lateral signaling interactions between CD47 and other membrane signaling proteins including integrins and tyrosine kinase receptors. Specific CD47 antibodies directly induced death of malignant cells (Kikuchi et al., 2005; Puro et al., 2020), pancreatic cancer stem cells (CSC) (Cioffi et al., 2015), and sensitized malignant cells to chemotherapy (Lo et al., 2015). CD47 antibodies induced a caspase-independent cell death pathway in breast cancer (Manna and Frazier, 2004) and leukemia cells (Mateo et al., 1999). CD47 antibody treatment or CD47 knockdown suppressed stem cell character in hepatocarcinoma, MDA-MB-231 breast carcinoma, and glioma cells (Lee et al., 2014; Soto-Pantoja et al., 2015; Li et al., 2018; Tan et al., 2019).

Cancer stem cells and their biomarkers are potent targets to restrain chemoresistance, metastasis, and tumor relapse by limiting capacity of self-renewal and differentiation of cancer cells (Raghav and Mann, 2021). We found that treatment with the CD47 antibody B6H12 altered gene expression in CD47-expressing triple-negative MDA-MB-231 breast carcinoma cells, resulting in suppression of breast cancer stem cell (bCSC) characteristics (Kaur et al., 2016). B6H12 treatment decreased expression of Klf4 (Kaur et al., 2016), one of several stem cell transcription factors that are also regulated by TSP1/CD47 signaling in nonmalignant cells (Kaur et al., 2013). B6H12 inhibited asymmetric division of breast cancer stem cells and induced several differentiation markers. Notably, B6H12 treatment of bCSCs derived from MDA-MB-231 cells increased their expression of miR-7 and decreased EGFR mRNA, which is a target of miR-7. These studies suggest that therapeutic CD47 antibodies and SIRPα decoys intended to block SIRPα signaling in innate immune cells could also alter CD47 signaling in cancer cells in a manner that provides therapeutic benefits. In the present study, we tested direct effects on MDA-MB-231 cells of divalent recombinant SIRPα-Fc (SIRPFc) and the humanized CD47 antibody CC90002, which selectively inhibits the CD47-SIRPα interaction (Pau Abrisqueta, 2019; Narla et al., 2022; Zeidan et al., 2022).

## 2 Materials and methods

### 2.1 Cell culture

Triple negative MDA-MB-231 breast carcinoma cells were purchased from ATCC and were routinely cultured in RPMI 1640 (Invitrogen, 11875-093) medium supplemented with 10% FBS (Gemini Bio Products, GBP-100106), penicillin-streptomycin (Gibco, catalog number #10378016/15070-063) and L-glutamine (Catalog number # A2916801 complete growth medium (Thermo Fisher Scientific, USA) at 37°C and 5% CO_2_ as reported previously (Kaur et al., 2016).

### 2.2 SIRPα-Fc purification

HEK 293 cells were cultured using DMEM complete media with 10% FBS, Penicillin-Streptomycin, and L-glutamine at 37° with 5% CO_2_. SIRPα-Fc plasmid DNA ((Miller et al., 2019) was transfected in to HEK293 cells using DMEM serum free media with 0.01%BSA. After 2 days, the media were collected and concentrated using Millipore Sigma Amicon Ultra-15 Centrifugal Filter Units (Fisher Scientific, USA) with a 10 kDa cutoff. Concentrated condition media were purified via Protein A IgG Purification Kit (Thermo Fisher Scientific, USA). Purity of the SIRPα-Fc was confirmed using gel electrophoresis, and concentration was determined using the BCA assay (Thermo Fisher Scientific). For some experiments SIRPα-Fc was purchased from R&D systems. Monovalent SIRPα-Avi-Biotin (SIRPmv) was used as reported previously (Miller et al., 2019).

### 2.3 Flow cytometry analysis

MDA-MB-231 cells were pretreated with the Celgene antibody for times indicated in the Figure Legends. MDA-MB-231 Cells were then stained with PE anti-human CD47, APC/Cy7 anti-human CD24, FITC anti-human CD44 along with isotype control antibodies; FITC Mouse IgG1, PE Mouse IgG1, APC/Cy7 IgG2a (Biolegend, USA). MDA-MB-231 cells were washed three times and resuspended in Hanks’ balanced salt solution at 1 × 10^6^ cells in 500 μL. Samples then were acquired on a LSRII (BD Biosciences). Flow cytometry data were analyzed using FlowJo software (TreeStar).

### 2.4 Cancer stem cell markers analysis

bCSC were generated using sorted CD44^+^/CD24^-^ (Supplementary Figure S1F) cells from the MD-MB-231 cell line. The suspension cells were cultured using SmartDish™ with MammoCult™ Human Medium Kit from Stem Cell Technologies. The suspension cells were treated with CC90002 (1 μg/mL) or SIRPα-Fc (1 μg/mL) for 72 h, and analyzed using APC/Cy7 anti-human CD24, FITC anti-human CD44 along with FITC Mouse IgG1, κ isotype Ctrl, κ Isotype Ctrl (FC), APC/Cy7 IgG2a, κ isotype Control flow antibodies. For mRNA expression, suspension cells were generated from MDA-MB-231 by using AggreWell™400 with AggreWell™ EB Formation Medium from Stem Cell Technologies and treated with CC90002 (1 μg/mL) and SIRPα-Fc (1 μg/mL) for 72 h as described above.

### 2.5 CD24^High^ and CD24^Low^ subset enrichment

MDA-MB-231 cells were washed with PBS, dissociated with Gentle cell dissociation Reagent (STEM Cell Technologies) and centrifuged for 5 min at 1,200 rpm. The cells were re-suspended into cell isolation buffer containing phosphate-buffered saline (PBS), pH 7.2, 0.5% bovine serum albumin (BSA), and 2 mM EDTA. MDA-MB-231 cells were incubated with biotin anti-human CD24 (Biolegend) for 10 min on ice. Mojosort™ Streptavidin Nanobeads were added to the respective tubes containing cells and incubated for another 10 min on ice. Enriched CSCs cells were separated using MiniMac Separation columns, type MS (Miltenyi Biotech Inc.) according to the manufacturer’s instructions. The CD24^high^ (CD24^+^) cells became attached to beads, while the CD24^Low^ cells (CD24^−^) flowed through the columns and were collected into new tubes as shown in Supplementary Figure S1G via flow cytometry analysis. The sorted CD24^+^ and CD24^−^ cells were further treated with divalent or monovalent SIRPα-Fc for 24 h, and total RNA was extracted for Real Time PCR analysis.

### 2.6 Real Real Time PCR

Total RNA was isolated using either TRIzol Reagent (Thermo Fisher Scientific) or TriPure™ Isolation Reagent from Roche (Millipore Sigma). cDNA was synthesized using the Maxima First Strand cDNA Synthesis Kit for RT-qPCR, with dsDNase (Thermo Fisher Scientific) Quantitative real-time PCR was performed using KLF4, OCT4, SOX2 using 18S or B2M, SNAIL, SLUG, ZEB1, ZO-1, ALDH1A1, CDH1, CTNNB1 and CSNK1A1 primers using SYBR Green (Thermo Fisher Scientific) on an MJ Research Opticon I instrument (Bio-Rad) as control as described previously (kaur et al., 2019, scientific reports). EMT and cancer stem cell primers (Table 1) were purchased from Integrated DNA Technologies (IDT, USA). The results were quantified as Ct values and expressed as fold gene expression (the ratio of treated/control).

**TABLE 1:** Primer sequences used for RT-qPCR analysis of mRNA expression for the indicated genes.

Name	Primer sequence
snail-F	AGG​CCA​AGG​ATC​TCC​AGG​CTC​GA
snail-R	CTT​CCC​GCA​GGT​TCC​GCA​GA
SLUG-F	TGC​ACT​GCG​ATG​CCC​AGT​CT
SLUG-R	AAA​ACG​CCT​TGC​CGC​AGA​TC
β_Catenin-F	AAG​TCT​GGA​GGC​ATT​CCT​GC
β_Catenin-R	ACC​AGC​TAA​ACG​CAC​TGC​CA
ZEB1-F	CGC​TTC​TCA​CAC​TCT​GGG​TC
ZEB1-R	CAT​TCG​AGA​GGA​TTT​CAG​GCC​CT
ZO-1-F	CGT​TAG​TCA​CCC​AGG​GCA​CAG​G
ZO-1-R	GTA​TGT​GGG​CTG​CTC​GAG​GT
B2M-F	TCC TGA ATT GCT ATG TGT CTG GGT
B2M-R	GAT AGA AAG ACC AGT CCT TGC T
18S-F	AGG ACC GCG GTT CTA TTT TGT TGG
18S-R	CCC CCG GCC GTC CCT CTT A
ALDH1A1-F	CCA CTC ACT GAA TCA TGC CA
ALDH1A1-R	GCA CGC CAG ACT TAC CTG TC
CK2-F	AGC ATG CCA GGG GGC AGT AC
CK2-R	CTG GTG AGC CTG CCA GAG GT
CDH1-F	CCA GAA TCC CCA AGT GCC TGC
CDH1-R	GAA TTG GGC AAA TGT GTT CAG C

### 2.7 Aldefluor assay

MDA-MB-231 cells were cultured in AggreWell™ Embryoid Body (EB) Formation Medium using SmartDish™ (STEMCELL Technologies) and treated with CC90002 (1 μg/mL) and SIRPα-Fc (1 μg/mL) alone for 72 h at 37°. The loosely aggregated spheroids were suspended into single cells and subjected to BODIPY-aminoacetaldehyde (BAAA) aldehyde dehydrogenase substrate and/or DEAB as negative control staining using ALDEFLUOR™ kit from STEMCELL Technologies. The protocol and flowcytometry analysis were followed according to manufacturer’s instructions. % ALDH^br^ cells from three independent experiments were calculated using background subtraction of negative control.

### 2.8 Cell proliferation assays

IncuCyte NucLight for cell nuclear labeling (ESS4717, Essen BioScience) was used for living cells. MDA-MB-231 (∼1,000 cells/50 µL) were plated in a 96-well plate. CC90002 antibody (1 μg/mL) and SIRPα-Fc (1 μg/mL) were added alone or in combination. Diluted IncuCyte Nuclight Rapid live cell labeling reagent (50 µL) added to each well according to manufactures instructions (Essen BioScience, Santorious). The cells were analyzed every 4 h for 7 days using Basic Analyzer analysis (IncuCyte system software).

### 2.9 Spheroid/mammosphere assay

MDA-231 cells were grown in culture, (RPMI media w/1% Pen-Strep, 1% L-glutamine, and 10% fetal bovine serum at 37° with 5% CO_2_. Approximately 1000 MDA-MB-231 cells were transferred to a small petri dish along with AggreWell™ Embryoid Body (EB) Formation Medium or MammoCult™ Human Medium Kit and treated with CC90002 (1 μg/mL) or SIRPα-Fc (1 μg/mL) alone for 10 days at 37°. The total number of mammospheres were counted using a light microscope, and number of spheroids were plotted using Prism software. For live cell mammospheres formation, MDA-MB-231 triple-negative breast carcinoma cells were labeled with PKH26 dye (Sigma) at day 0 according to the manufacturer’s instructions. Approximately 1,000 cells were transferred into each well of a 96-well plate using mamo cult media. Images were captured every 4 h using Spheroid analysis with the IncuCyte system software.

### 2.10 SIRPα-CD47 binding assay markers analysis

MDA-MB-231 cells were trypsinzed, and expression of CD47 and SIRP**α** were measured using flow cytometry (Supplementary Figure S1A, B). To investigate effects of CC90002 Ab on SIRP**α**-CD47 binding, cells were incubated with monovalent SIRP-**α-**biotin (Miller et al., 2019) on ice for 30 min in the presence or absence of CD47 antibodies. The expression of SIRP**α** was analyzed using streptavidin AF-488 via flow cytometry analysis.

### 2.11 Bulk RNA sequencing analysis

MDA-MB-231 cells were treated CC90002 (1 μg/mL) and SIRPα-Fc (1 μg/mL) alone for 72 h at 37° along with untreated and human isotype control (Celgene) using AggreWell media (Stem Cell Technologies) according to manufacturer’s instructions. Total RNA was extracted using ISOLATE II RNA Mini Kit from BIOLINE, and RNA integrity and quantification was measured using RNA-Bioanalyzer.

All mRNA-seq samples were pooled and sequenced on NextSeq using Illumina TruSeq Stranded mRNA Library Prep and paired-end sequencing (GEO Accession Number: GSE247052). The samples had 20 to 33 million pass filter reads with a base call quality of above 83% of bases with Q30 and above. Reads of the samples were trimmed for adapters and low-quality bases using Trimmomatic software before alignment with the reference genome (Human - hg38) and the annotated transcripts using STAR. The average mapping rate of all samples was 95%. Unique alignment is above 57%. There were 1.81%–38.56% unmapped reads. The mapping statistics were calculated using Picard software. The samples had 1.04% ribosomal bases. Percent coding bases are between 44% and 60%. Percent UTR bases are 31%–42%, and mRNA bases were between 79% and 92% for all the samples. Library complexity was measured in terms of unique fragments in the mapped reads using Picard’s Mark Duplicate utility. The samples had 59%–91% non-duplicate reads. In addition, the gene expression quantification analysis was performed for all samples using STAR/RSEM tools.

The RNA-Seek pipeline (https://github.com/CCBR/RNA-seek) was used to process reads. Expected counts from RSEM for both genes were imported into the NIH Integrated Data Analysis Platform (Palantir Technologies) for downstream analysis as described earlier (Nath et al., 2022). Briefly, low count genes were filtered prior to CPM and quantile normalization using Limma voom (Smyth, 2004; Law et al., 2014), followed by differential expression of genes analysis. Pre ranked gene set enrichment analysis (GSEA) using molecular signatures database v6.2 (Subramanian et al., 2005; Liberzon et al., 2011). Batch correction was performed using the ComBat function of the sva (Johnson et al., 2007).

### 2.12 Statistics

All experiments were replicated at least three times on different groups of cells. All data are expressed as mean ± SD. The differences were considered significant at *p* values <0.05 as indicated in the figure legends.

## 3 Results

### 3.1 Direct effects of SIRPα-Fc and CC90002 on MDA-MB-231 cells

**FIGURE 1:**
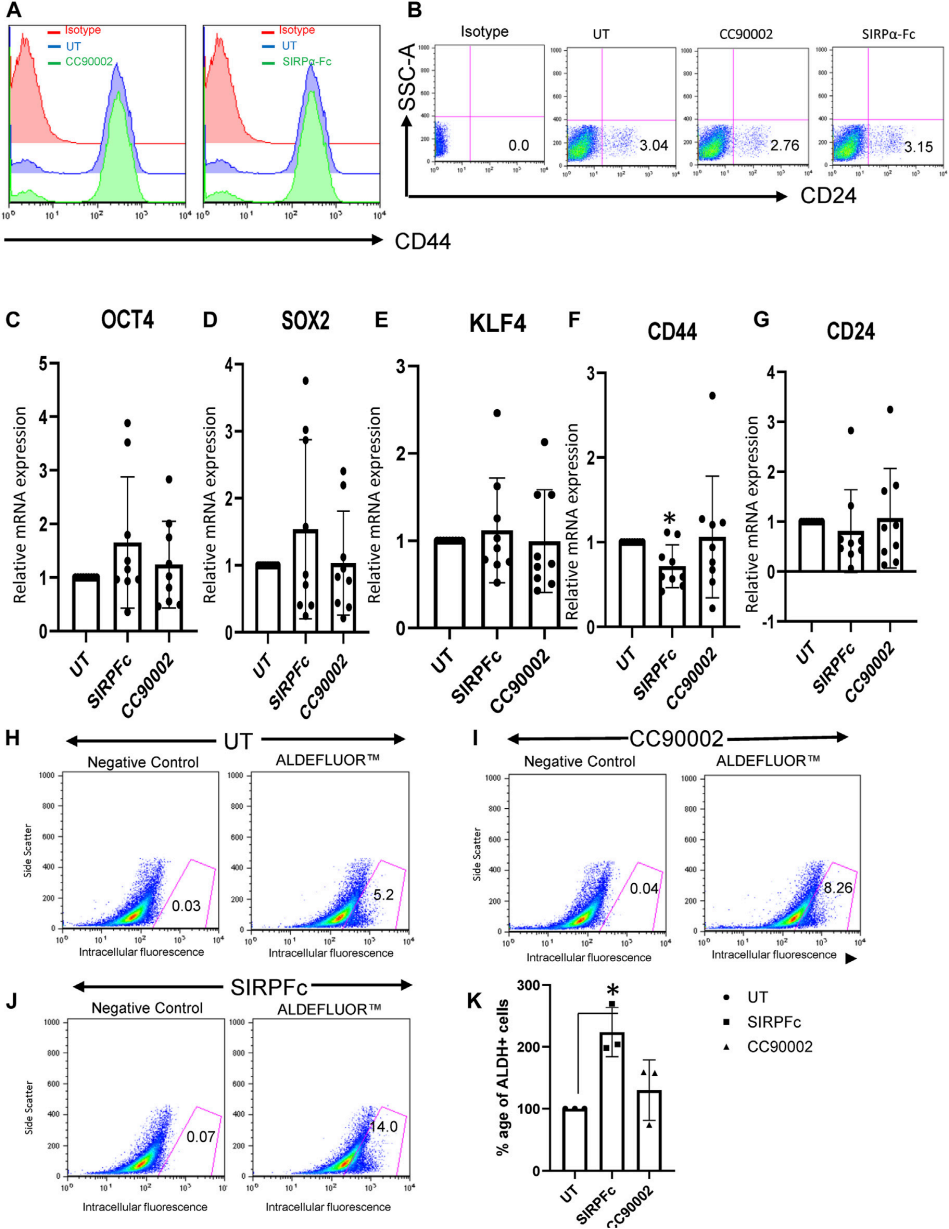
SIRPα-Fc increased Aldefluor activity in MDA-MB-231 cells. **(A, B)** MDA-MB-231 cells were grown and treated with CC90002 **(C–G)**, 1 μg/mL) and SIRPα-Fc (1 μg/mL) for 72 h. The total cells were harvested, and flow analysis was performed using anti-CD24 and CD44 antibodies. **(C–G)** MDA-MB-231 cells were grown in AggreWell embryoid formation media and treated with CC90002 (1 μg/mL), SIRPα-Fc (1 μg/mL) for 72 h. The total RNA was extracted using miRNA easy kit, and OCT4, SOX2, KLF4, CD44 and CD24 mRNA expression were analyzed via qPCR (n = 3, 3 replicates for each experiment). **(H–J)** FACS profiles of DEAB control and ALDH1 staining with flow cytometry (n = 3 independent experiments). **(K)** graph showing the percentage of ALDH1^+^ cells following treatment using CC90002 (1 μg/mL) or SIRPα-Fc (1 μg/mL). Significant values (*p* < 0.05) were calculated using a t-test.

Based on our previous studies demonstrating effects of the CD47 antibody B6H12 on stem cell markers in MDA-MB-231 cells (Kaur et al., 2016), which have a high number of bCSC as compared to MCF7 or T47D1 cell lines (Honeth et al., 2008), we examined effects of SIRPα-Fc and the CC90002 antibody on the expression of CD44 and CD24. Flow cytometry confirmed that MDA-MB-231 cells highly express cell surface CD47, whereas only a minor population express detectable SIRPα (Supplementary Figure S1A, B). Binding of SIRPα-Fc to cell surface CD47 was inhibited in the presence of CC90002 (Supplementary Figure S1C). However, treatment of MDA-MB-231 cells with SIRPα-Fc (SIRPFc) or CC90002 (CG) for 72 h did not significantly alter cell surface expression of the stem cell markers CD44 or CD24 (Figures 1A, B). Consistent with these results, treatment of MDA-MB-231 cells with 1 μg/mL of SIRPα-Fc or the CC90002 antibody for 72 h did not significantly change the mRNA expression of *OCT4, SOX2, KLF4, CD44*, and *CD24* genes. However*,* SIRPα-Fc treatment significantly downregulated CD44 mRNA (Figures 1C–G). Therefore, the effects of SIRPα-Fc and CC90002 antibody on mRNA expression of pluripotent stem cell markers differ from those of B6H12.

Aldehyde dehydrogenase 1 (ALDHA1) is a CSC marker expressed in basal breast cancers (Tan et al., 2013). Expression of specific isoforms of (ALDHA1) and the corresponding enzymatic activity are useful markers of CSC differentiation and can be quantified by flow cytometry using Aldefuor (Moreb, 2008; Marcato et al., 2011; Tomita et al., 2016). MDA-MB-231 cells cultured on plastic in RPMI medium had undetectable ALDHA1 mRNA (Supplementary Figure S1D). MDA-MB-231 cells cultured in AggreWell medium exhibited expression of ALDHA1, but treatment with CC90002 or SIRPα-Fc did not significantly change expression of ALDHA1 mRNA (Supplementary Figure S1E). However, assessment of ALDH1 activity using the ALDEFLUOR Kit, indicated that, SIRPα-Fc significantly increased the percentage of Aldefluor-positive cells as compared to untreated (Figure 1H, J, K). Treatment with CC90002 resulted in a smaller increase in Aldefluor-positive cells that was not statistically significant (Figure 1I, K). Therefore, SIRPα-Fc may be a selective CD47-dependent inducer of stem cell character in these breast carcinoma cells.

### 3.2 Effect of SIRPα-Fc on cell proliferation and spheroid formation

**FIGURE 2:**
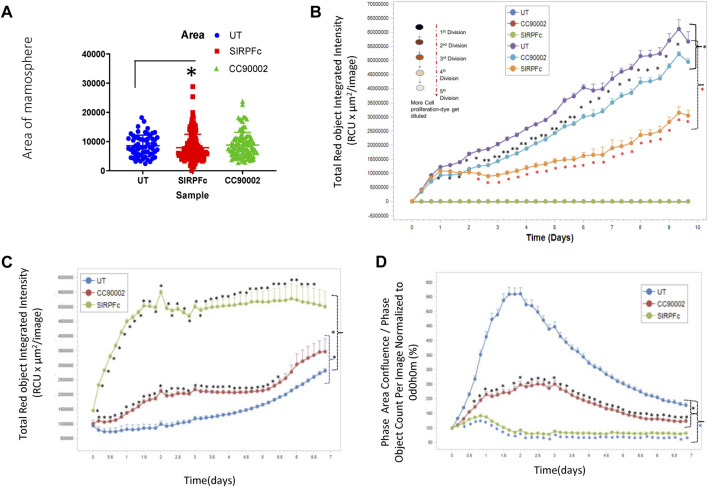
**(A)** SIRPα-Fc increased spheroid formation in MDA-MB-231 cells. MDA-MB-231 cells (∼1,000) were cultured using smart dish in 2 mL of AggreWell medium. Cells were treated with CC90002 (1 μg/mL) and SIRPα-Fc (1 μg/mL). alone for 10 days. The total number of mammospheres were counted using a light microscope. The results were then plotted using Prism. Significant values (*p* < 0.05) were calculated using F-Test Two-Sample for Variances. **(B)** MDA-MB-231 cells were labeled with PKH26 dye (Sigma) on day 0 (n = 4). Approximately 1,000 cells/well were plated in 96-well plates (n = 4 replicates/treatments) and treated with CC90002 (1 μg/mL) or SIRPα-Fc (1 μg/mL). Images were captured every 4 h for 10 days using the system software as indicated in graphs. Significant values (*p* < 0.05) were calculated using t-test for two samples assuming equal variance. **(C)** Cell proliferation of MDA-MB-231 cells was determined after labeling with Rapid Red dye using phase contrast imaging **(D)** in the presence of CC90002 (1 μg/mL) or SIRPα-Fc (1 μg/mL) as described above. Significant values were determined using multiple t-tests.

Formation of mammospheres provides a quantitative assessment of stem cell character and aggressiveness in breast carcinoma cells (Shaw et al., 2012; Margaryan et al., 2019). SIRPα-Fc treatment resulted in ∼3-fold increase in the number of mammospheres and their combined area (Figure 2A). The area of mammospheres increased less in cultures treated with CC90002 antibody and did not achieve significance. This data indicates that SIRPα-Fc increases either the proliferation or self-renewal capacity of breast cancer stem cells (Figure 2A). We also assessed Mammosphere size using a spheroid dye dilution assay on the IncuCyte instrument (Figure 2B) along with unlabeled control treatments. Depletion of the PKH dye signal indicated increased cell proliferation in spheroids. Decreased fluorescence in SIRPα-Fc-treated cells indicated strong stimulation of cell proliferation, whereas the CC90002 antibody had minimal activity (Figure 2B). SIRPα-Fc (1 μg/mL) treatment increased mammosphere size over 5 days, whereas CC90002 antibody was less active as shown in Figure 2A.

To assess effects of CD47 ligands more directly on MDA-MB-231 bCSC proliferation, we used the IncuCyte Rapid Red Dye assay (Figure 2C). SIRPα-Fc treatment of MDA-MB-231 cells resulted in the most increase in cell growth. CC90002 modestly increased cell proliferation of MDA-MB-231 cells.

### 3.3 SIRPα-Fc affects expression of EMT markers

To examine effects of these CD47 ligands on the global transcriptome, MDA-MB-231 cells were grown in AggreWell Embryoid Body (EB) Formation media for 72 h in the presence of SIRPα-Fc (1 μg/mL), CC90002 (1 μg/mL), IgG isotype antibody control, or untreated controls (n = 3). Bulk RNA sequencing analysis was performed and analyzed using the NIDAP platform. The QC with Batch Correction, Differential Expression of Genes and Visualization data indicated consistent high-quality results (Supplementary Figure S2A-C). Differentially expressed genes between SIRPα-Fc and CC90002 antibody treatments compared with untreated or control IgG treatment were determined using a threshold *p*-value of 0.05 (Supplementary Figure S2D). The differentially expressed genes between SIRPα-Fc *versus* UT, and SIRPα-Fc *versus* IgG or CC90002 *versus* UT and CC90002 *versus* IgG comparisons are shown in Supplementary Data S1. Gene set enrichment analysis (GSEA) for multiple pathways (Supplementary Figure S2E) and list of significant GSEA datasets is summarized in Supplementary Data S2.

**FIGURE 3:**
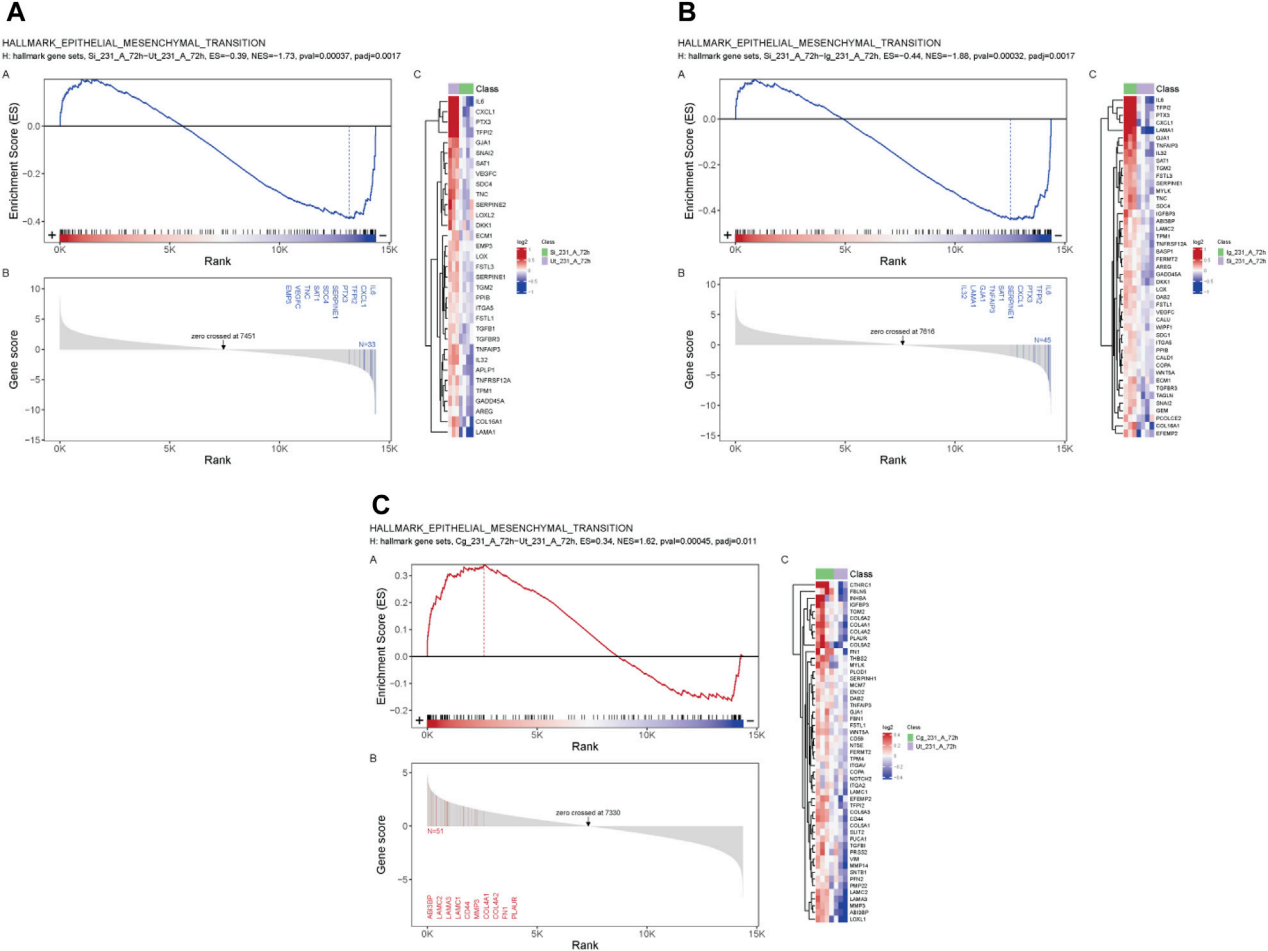
SIRPα-Fc negatively correlated with Epithelial Mesenchymal transition in MDA-MB-231 cells. MDA-MB-231 cells were grown in AggreWell media, treated with CC90002 antibody (1 μg/mL) or SIRPα- Fc (1 μg/mL) along with control IgG or untreated (n = 3). After 72 h, total RNAs were extracted, and mRNA sequencing analysis was performed using the NIDAP platform. GSEA plots showing differential expressed genes **(A, B)** SIRPFc Vs. UT and SIRPFc Vs. IgG and, **(C)** CC90002 Vs. UT GSEA-plots using msigdb_v6_2_with_orthologs show enrichment Hallmark of Epithelial Mesenchymal transition.

GSEA revealed a negative enrichment between SIRPα-Fc *versus* untreated, and SIRPα-Fc *versus* IgG treatments (Figures 3A, B, ES = −0.39, *p*-value 0.00037 and ES = −0.44, pval 0.00032) for genes that regulate epithelial mesenchymal transition (EMT), whereas CC90002 treatment showed a positive enrichment of the same gene set (ES = 0.34) (Figure 3C), which was significant compared to untreated samples but not significant for Isotype control antibody treated (Data S2).

**FIGURE 4:**
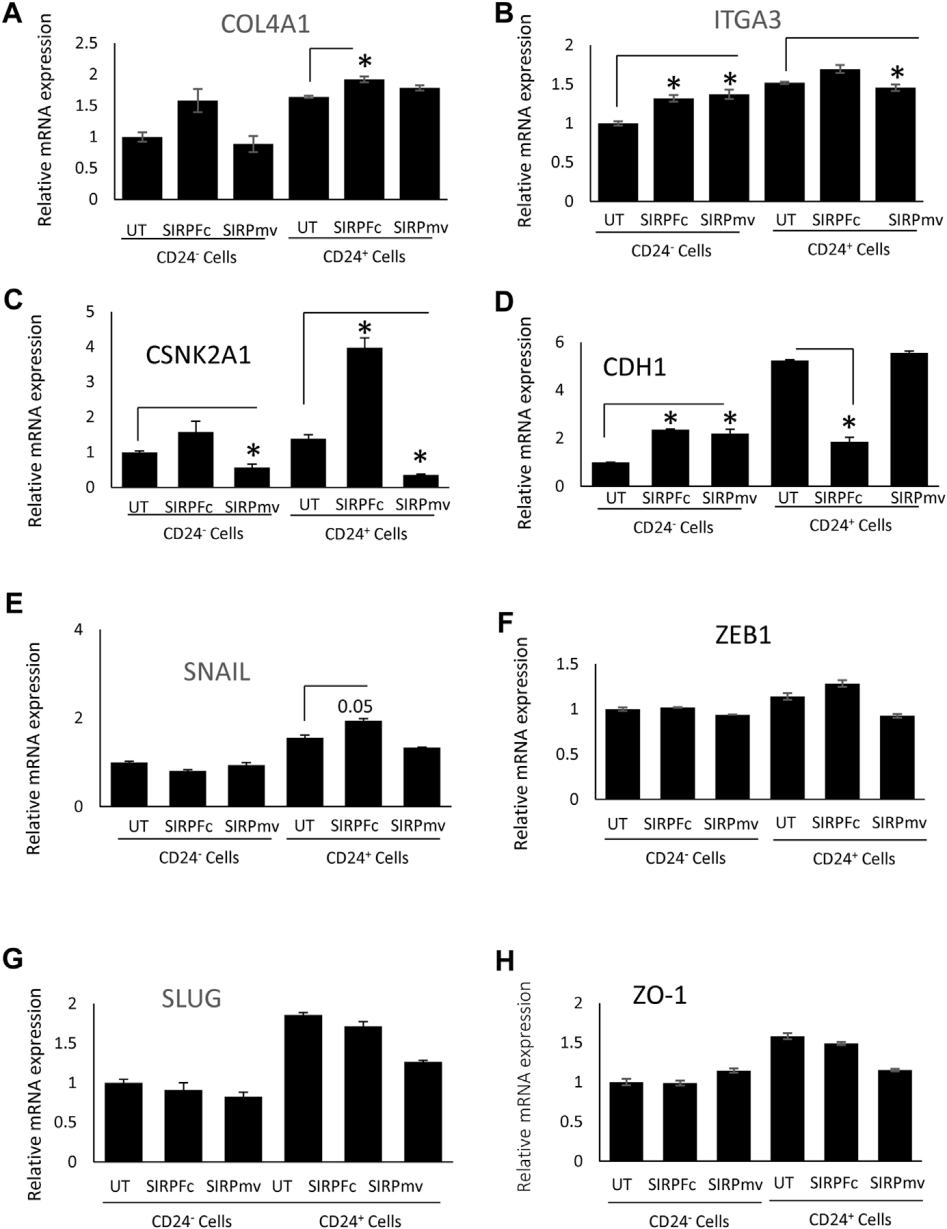
Differential effect of SIRPα-Fc or SIRPmv on expression of EMT markers using CD24^−^ or CD24^+^ subsets derived from MDA-MB-231 cells **(A–H)** Expression of EMT markers were analyzed using SIRPα-Fc divalent or monovalent SIRPmv via q-PCR analysis (n = 2 replicates per treatments). Significant values (*p* > 0.05) were calculated with ∆CT using Anova: Single Factor by comparing SIRPFc divalent or monovalent SIRPmv treated with either Untreated CD24^−^ or CD24^+^ cells.

The stem cell marker CD24 is associated with EMT and cell proliferation (Nakamura et al., 2017) and has additive effects with CD47 to regulate phagocytosis (Ozawa et al., 2021). Therefore, we examined expression of genes in this pathway in CD24^+^ enriched and CD24^−^ MDA-MB-231 cells (Figures 4A–H). COL4A1 and ITGA3 genes were selected from list of GSEA Figures 3A, B to validate the RNA sequencing analysis based on their role in proliferation, EMT and stemness of breast cancer cells (Halsted et al., 2008; Jin et al., 2017; Wang et al., 2020b; Zhang et al., 2020). SIRPα-Fc but not SIRPmv treatment significantly enhanced expression of COL4A1 mRNA in CD24^+^ cells but not in CD24^−^ cells, whereas ITGA3 mRNA expression was significantly upregulated in CD24^−^ cells and decreased in CD24^+^ cells with SIRPmv treatment (Figures 4A, B). Treatment using divalent SIRPα-Fc significantly increased casein kinase 2 (CK2/CSNK2A1) mRNA levels in CD24^+^ cells but only modestly in CD24^−^ cells. In contrast, treatment using monovalent SIRPmv significantly downregulated casein kinase 2 mRNA in both CD24^+^ and CD24^−^ cells (Figure 4C). These divergent responses suggest that dimerization of cell surface CD47 by SIRPα-Fc results in a different signal than monovalent ligation of CD47 by SIRPmv. SIRPα-Fc but not SIRPmv treatment downregulated E-cadherin (CDH1) mRNA expression in CD24^+^ cells, but modestly increased expression in CD24^−^ cells (Figure 4D). Similarly, SNAIL mRNA was modestly upregulated with SIRPα-Fc but not SIRPmv treatment only in CD24^+^ cells (Figure 4E). The EMT markers ZEB1, SLUG and ZO-1 were not significantly changed with SIRPα-Fc or SIRPmv treatments in both CD24^−^ and CD24^+^ cells (Figures 4F–H).

**FIGURE 5:**
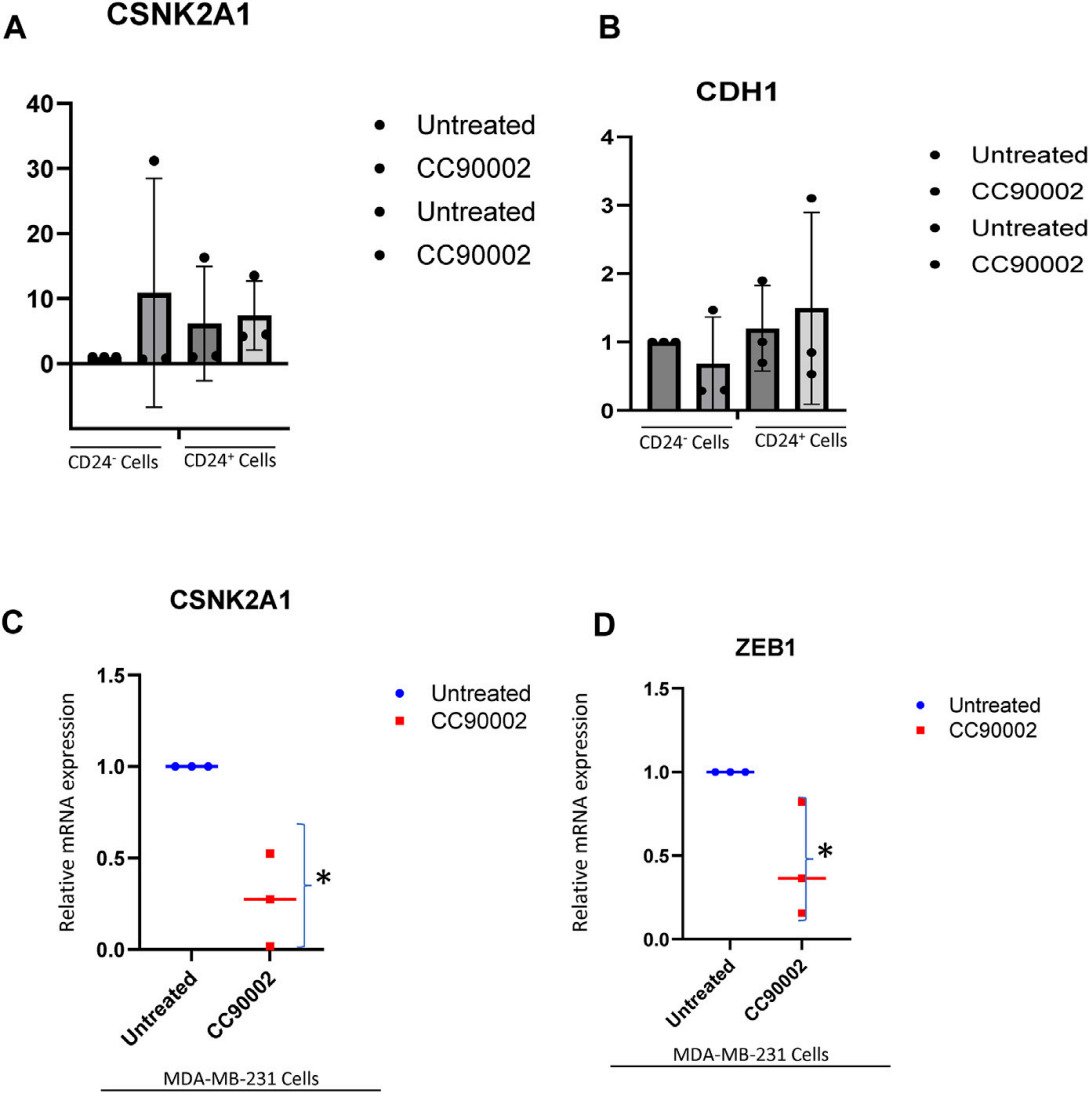
Differential effect of CC90002 on expression of EMT markers using MDA-MB-231 cells. **(A, B)** Differential effect of CC90002 (1 μg/mL) on expression of CK2 and CDH1 markers using CD24^−^ or CD24^+^ subsets derived from MDA-MB-231 cells via q-PCR analysis (n = 3, 3replicates per treatments). Significant values (*p* < 0.05) were calculated using default setting of CFX Mastro (BioRad)software by comparing CG treated with either Untreated CD24^−^ or reconfirmed using GraphPad Prism 10.02 two-tailed t-test. **(C, D)** MDA-MB-231 cells were grown in AggreWell media, treated with CC90002 antibody (1 μg/mL) along with untreated (n = 3). After 7 2 h, total RNAs were extracted, and CK2 and ZEB1 mRNA expression were analyzed. Significant values (*p* < 0.05) were calculated using two-tailed t-test by using default setting of GraphPad Prism 10.02.

In contrast to SIRPα-Fc. CC90002 antibody treatment had no significant effect on CK2 and CDH1 mRNA expression in CD24^+^ and CD24^−^ cells (Figures 5A, B). However, using unfractionated MDA-MB-231 cells, CC90002 antibody treatment significantly downregulated CK2 and ZEB1 mRNA expression (Figures 5C, D), but no other EMT markers were significantly altered (Supplementary Figure S3A-C). Loss of E-cadherin has been associated with progression and survival in human breast cancer (Lipponen et al., 1994; Singhai et al., 2011). This may indicate that SIRPα-Fc treatment increases cancer cell survival, and CK2 may be a key player for EMT driven proliferation or spheroid formation (Zhang et al., 2014). However, the effects of CC90002 diverged from those of SIRPα-Fc, indicating that effects of therapeutic CD47 binding molecules on breast cancer stem cells are ligand-specific.

## 4 Discussion

Comparing the present data with our previous study of the effects of the CD47 antibody B6H12 on the same cells indicates that different CD47 antibodies can have divergent effects on CD47 signaling in triple-negative breast cancer cells. Although both B6H12 and the humanized antibody CC90002 inhibit CD47 binding to SIRPα, each has different effects on MDA-MB-231 cells. A small fraction of MDA-MB-231 cells express SIRPα at a level detectable by flow cytometry. Thus, some of activities of these agents may involve blocking cis-interactions between CD47 and SIRPα, but this cannot explain why the activities of monovalent and divalent soluble SIRPα-Fc have divergent effects on gene expression.

Our data suggests that therapeutics based on SIRPα Ig domains that bind to CD47 have the potential to enhance cancer stemness in triple negative breast cancer cells as evidenced by increasing spheroid formation, cell proliferation and ALDH1 expression, which is a universal marker for stem and progenitor cells and also associated with poor prognosis in triple negative breast cancer (Panigoro et al., 2020). Thus, the benefit of these therapeutics to increase phagocytic clearance may be offset by increased resistance to cancer treatments by altering expression of EMT markers (Luo and Yao, 2014; Pai et al., 2019). Downregulation of CDH1 mRNA expression and increasing SNAIL and CSNK2A1 mRNA expression suggest that SIRPα-Fc treatment can increase the tumorigenic potential. Reduced E-cadherin expression in invasive breast carcinomas was correlated with triple negative receptor status (*p* = 0.0336), and poor prognosis (*p* = 0.0466) (Burandt et al., 2021). Loss of E-cadherin and upregulation of ALDH1 and EMT markers in breast cancer stem cells may play a role in tumorigenesis or metastatasis (Papadaki et al., 2014), which could have negative impacts on overall and progression-free survival in clinical trials using SIRPα-based therapeutics to treat triple negative breast cancer.

Further studies are needed to distinguish the potential agonist activities of CC90002 binding to CD47 from its established activity as an antagonist of SIRPα binding to CD47. Because spheroid formation involves increased cell-cell contacts, CC90002, in part could decrease spheroid formation by antagonizing intercellular CD47-dependent SIRPα signaling or SIRPα-dependent CD47 signaling. The CD47 antibody B6H12 prevents binding of SIRPα and TSP1 to CD47, and the latter interaction was shown to regulate stem cell differentiation (Kaur et al., 2013; Kaur et al., 2016). Unlike B6H12, our current data showed no inhibitory activity of CC90002 in expression of CD44, CD24 and KLF4. Therefore, the observed activities of CC90002 are unlikely to represent competitive inhibition of TSP1 signaling. However, the possibility remains that CC90002 could be a noncompetitive inhibitor or modulator of TSP1 signaling.

Because most MDA-MB-231 cells lack detectable levels of SIRPα, antagonism of cis- or cell-cell SIRPα signaling through CD47 should have limited relevance to proliferation and gene expression changes that are independent of spheroid. Therefore, the most likely mechanism involves a direct agonist activity of CC90002. Two mechanisms could contribute to an agonist activity of CC90002. First, published studies have implicated dimerization or clustering of CD47 in its functions (Kikuchi et al., 2005; Subramanian et al., 2006; Wang et al., 2020a), and several of the known lateral association partners of CD47 are known to initiate signals when dimerized (Wang et al., 2020c). Divalent SIRPα-Fc could increase dimerization or clustering of CD47, whereas SIRPmv could inhibit dimerization, which could account for some of the divergent gene expression changes we observed.

GSEA pathway analysis identified additional CD47 signaling pathways that are differentially regulated by CC90002 and SIRPα-Fc in MDA-MB-231 cells. identification of targets that are differentially regulated by SIRPα or TSP1 binding to CD47 will require further studies. This could identify additional beneficial therapeutic effects of CC90002 and SIRPα-Fc in malignant and nonmalignant cells as well as potential side effects that may be independent of its expected function as an antagonist of CD47-SIRPα signaling in phagocytes.

## Data Availability

The RNA sequencing data in this study are deposited in the Gene Expression Omnibus (GEO) database, accession number GSE247052. All original code has been deposited at Github (https://github.com/NIDAP-Community/Effects-of-a-humanized-CD47-antibody-and-recombinant-SIRPalpha-proteins-on-TNBC-stem-cells). Flow cytometry data are deposited on https://zenodo.org, DOI: 10.5281/zenodo10680474. Raw qPCR and IncuCyte data are available in the Supplementary Material.
