# Supplementary material for: Effects of a humanized CD47 antibody and recombinant SIRPα proteins on triple negative breast carcinoma stem cells
Source: Front Cell Dev Biol. 2024 Mar 1;12:1356421. doi: 10.3389/fcell.2024.1356421 (PMC10940465; doi:10.3389/fcell.2024.1356421)
Supplement: Supplementary file 3 [file Table1.DOCX]

Supplementary Material

**Effects of a humanized CD47 antibody and recombinant SIRPα proteins on triple negative breast carcinoma stem cells**

**Sukhbir Kaur^1^, Bianca Reginauld^1^, Sam Razjooyan^1^, Trung Phi ^1^, Satya P Singh^3^, Thomas J. Meyer^2^, Margaret C. Cam^2^, David D Roberts^1^**

1. ^Laboratory of Pathology, Center for Cancer Research, National Cancer Institute, National Institutes of Health, Bethesda, MD USA kaurs@mail.nih.gov; droberts@mail.nih.gov;^ [^biancareginauld@yahoo.co.uk^](mailto:biancareginauld@yahoo.co.uk)^;^ [^sam.razjooyan@aol.com^](mailto:sam.razjooyan@aol.com)^;^ ^trunghphi@gmail.com^
2. ^CCR Collaborative Bioinformatics, Resource, Office of Science and Technology Resources, National Cancer Institute, National Institutes of Health, Bethesda, MD, USA.^ [^margaret.cam@nih.gov^](mailto:margaret.cam@nih.gov)^;^ [^thomas.meyer@nih.gov^](mailto:thomas.meyer@nih.gov)
3. ^Inflammation Biology Section, Laboratory of Molecular Immunology, National Institute of Allergy and Infectious Diseases, National Institutes of Health, Bethesda, USA.^ [^spsingh@niaid.nih.gov^](mailto:spsingh@niaid.nih.gov)

*** Correspondence:** kaurs@mail.nih.gov (SK); droberts@mail.nih.gov (DDR)

# Supplementary Data

- DEG_Analysis_Final Data S1
- GSEA_Filtered_Final DATA S2

# Supplementary Figures and Tables

## Supplementary Figures


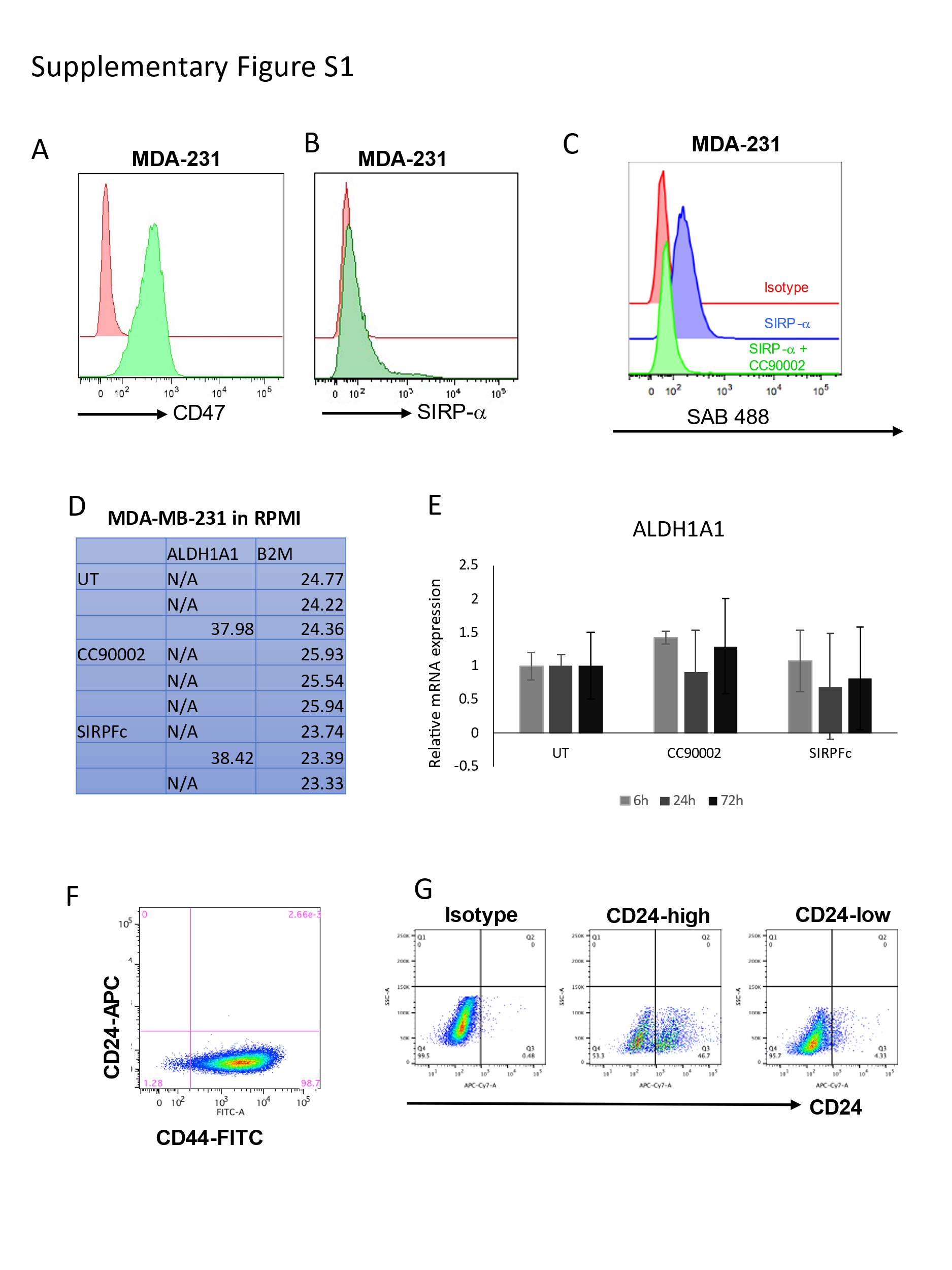


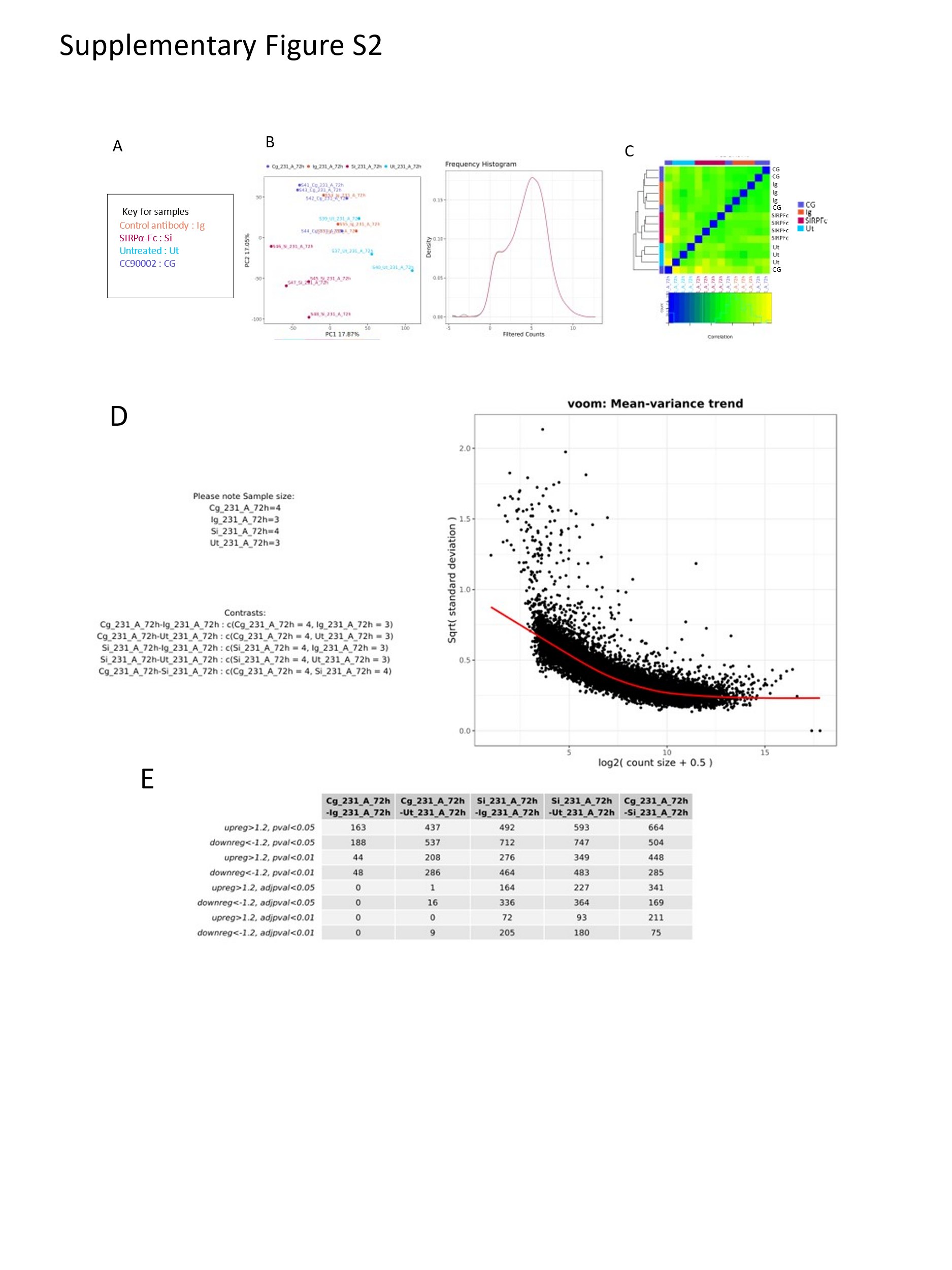


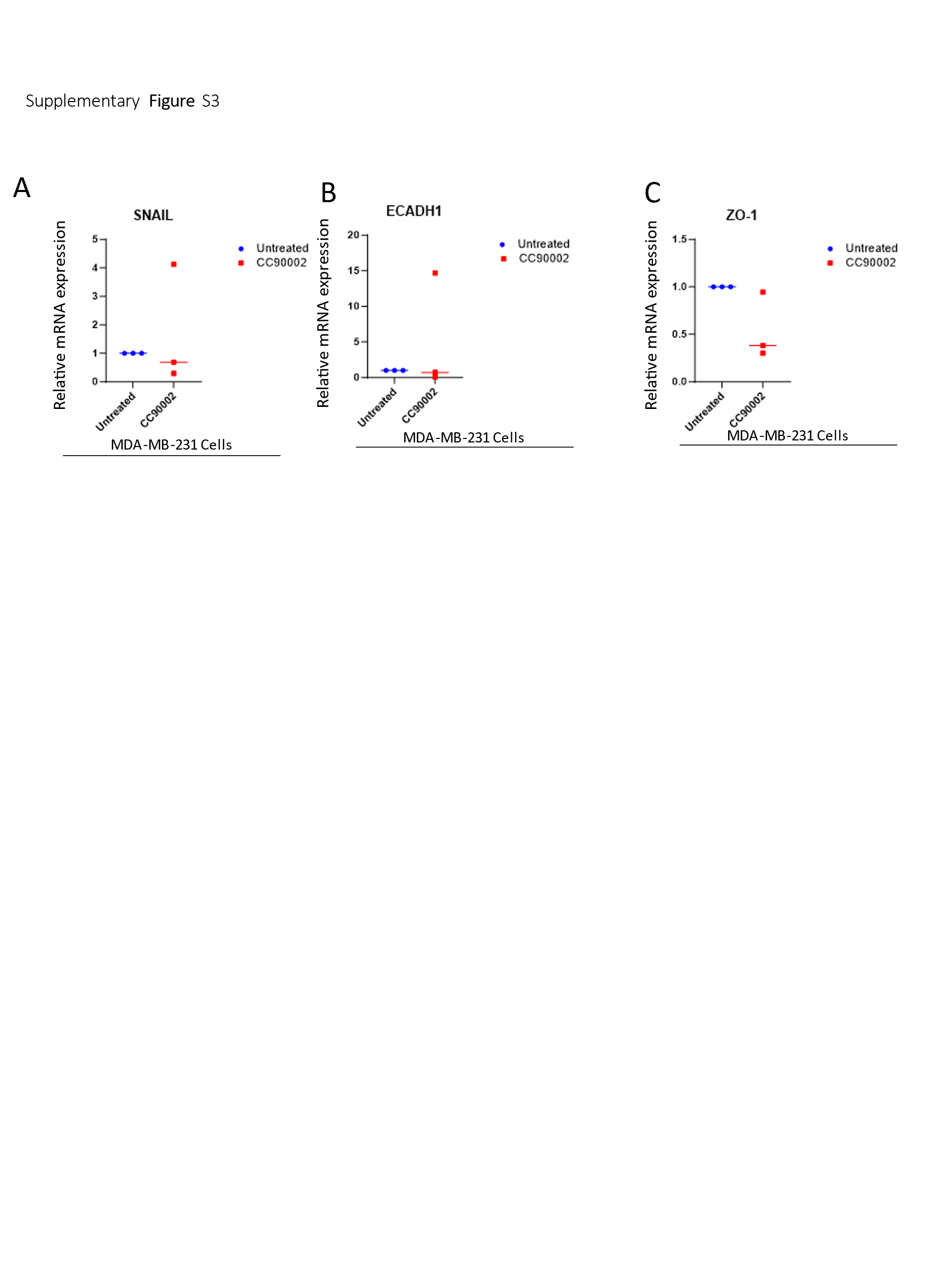


**
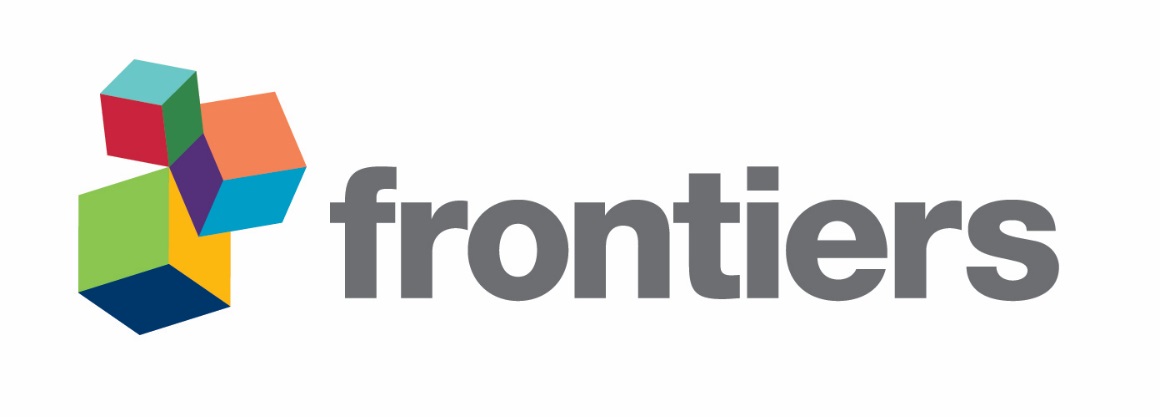
**

**Supplementary Figure Legends**

Figure S1: A) Expression of CD47 and SIRP alpha using MDA-MB-231 cells via flow cytometry analysis. C) CG disrupts binding between SIRPα and CD47. D&E) MDA-MB-231 cells were grown in either RPMI1640 or Aggrewell media for 72 hrs in the presence of CG or SIRPα-Fc. After 72 hrs, ALDH1 mRNA expression was analyzed via q-PCR (n=3). F) Sorted MDA-MB-231 cells were analyzed via flow cytometry analysis using anti-CD24 and CD44 antibodies. G) Purity of CD24+high and CD24-low Fractions of cells were determined using anti-CD24 antibody via flow cytometry analysis.

Figure S2: MDA-MB-231 cells were grown in Aggrewell media, treated with SIRPα-Fc or CG antibody (n=4) along with control IgG or untreated (n=3). After 72 hrs, total RNAs were extracted using the ISOLATE II RNA Mini Kit from BIOLINE, and mRNA sequencing analysis was performed using the NIDAP platform. A) Sample keys, B-C) PCA and QC plots, D& E) showing differential expressed genes.

Figure S3: A-C) Differential effect of CG on expression of EMT markers using MDA-MB-231 cells. MDA-MB-231 cells were grown in AggreWell™ EB Formation Medium, treated with CG antibody (1ug/ml) along with untreated (n=3). After 72hrs, total RNAs were extracted, and mRNA expression of SNAIL, ZO and CDH1 were analyzed. Significant values (p>0.05) were calculated using default setting of CFX Mastro software (BioRad) by comparing CG treated with Untreated cells or reconfirmed using GraphPad Prism 10.02 two-tailed t-test.
